# Draft Genome Sequences of the Onion Center Rot Pathogen *Pantoea ananatis* PA4 and Maize Brown Stalk Rot Pathogen *P. ananatis* BD442

**DOI:** 10.1128/genomeA.00750-14

**Published:** 2014-08-07

**Authors:** Tania Weller-Stuart, Wai Yin Chan, Teresa A. Coutinho, Stephanus N. Venter, Theo H. M. Smits, Brion Duffy, Teresa Goszczynska, Don A. Cowan, Pieter de Maayer

**Affiliations:** a Forestry and Agricultural Biotechnology Institute (FABI), Department of Microbiology, University of Pretoria, Pretoria, South Africa; b Environmental Genomics and Systems Biology Research Group, Institute for Natural Resources Sciences, Zurich University of Applied Sciences (ZHAW), Wädenswil, Switzerland; c Agricultural Research Council, Plant Protection Research Institute (ARC-PPRI), Pretoria, South Africa; d Centre of Microbial Ecology and Genomics (CMEG), University of Pretoria, Pretoria, South Africa

## Abstract

*Pantoea ananatis* is an emerging phytopathogen that infects a broad spectrum of plant hosts. Here, we present the genomes of two South African isolates, *P. ananatis* PA4, which causes center rot of onion, and BD442, isolated from brown stalk rot of maize.

## GENOME ANNOUNCEMENT

*Pantoea ananatis* is found in diverse natural environments and causes disease symptoms in a broad range of host plant species (1) including maize (2, 3), rice (4), and other economically important agricultural crops. *P. ananatis* was first isolated in South Africa from *Eucalyptus* seedlings displaying blight and dieback symptoms (5). Since then it has been isolated as the causative agent of brown stalk rot of maize in South Africa (3). It was also isolated from onion seeds and has been linked to center rot of this host (6). Here we report the draft genome sequences of two virulent *P. ananatis* strains isolated from maize (BD442) and onion seed (PA4) in South Africa. These strains were obtained from the Plant Pathogenic and Plant Protecting Bacterial Culture Collection, Agricultural Research Council–Plant Protection Institute, South Africa.

The genomes of *P. ananatis* BD442 and PA4 were sequenced using the Illumina HiSeq 2500 platform (2×51-bp shotgun sequencing). This yielded 63,960,136 (BD442) and 72,985,976 (PA4) paired-end reads representing an estimated coverage of 652× (BD442) and 744× (PA4), respectively. The genomes were assembled *de novo* using the Velvet short-read assembler plugin (7) of the Geneious Server (Biomatters, Ltd., Auckland, New Zealand) with approximately 16,000,000 reads per strain. Further gap closure was done by scaffolding the genomes against the complete *P. ananatis* clinical strain LMG5342 (8) and *Eucalyptus* strain LMG20103 (9) using Mauve version 2.3.1 (10).

The *P. ananatis* BD442 genome was assembled into eleven contigs, with a total size of 4.80 Mb, a mean G+C content of 53.59%, and an average contig length of ~436 kb, while that of PA4 was assembled into seventeen contigs, with a genome size of 5.16 Mb, a mean G+C content of 53.56%, and an average contig length of ~303 kb. Both assemblies incorporate complete circular plasmids, pPANA1BD442 (~353 kb; G+C%=51.13%) and pPANA1PA4 (~313 kb; G+C%=52.17%), that belong to the Large *Pantoea* plasmid-1 group, which plays a major role in the evolutionary diversification of *Pantoea* spp. (11). The genomes were annotated using the Rapid Annotations using Subsystems Technology (RAST) server (12). The genomes code for 4,673 (BD442) and 5,111 (PA4) proteins, respectively. Of these, 3,749 proteins are conserved between the two strains, while variability can largely be ascribed to prophage integration (13). We previously described three type VI secretion system (T6SS) loci in *P. ananatis* that play a role in animal and plant pathogenesis (14). All three loci (T6SS-1, -2, and -3) are present in *P. ananatis* PA4, whereas T6SS-3 is missing in BD442 (13, 14). These genomes will provide new insights into the pathogenic lifestyle of *Pantoea ananatis* and how it is able to cause disease symptoms on such a broad range of host plants.

### Nucleotide sequence accession numbers.

These whole-genome shotgun projects have been deposited in DDBJ/ENA/GenBank under the accession no. JMJK00000000 (*P. ananatis* PA4) and JMJL00000000 (*P. ananatis* BD442). The versions described in this paper are the first versions, JMJK01000000 (PA4) and JJML01000000 (BD442).
